# Myostatin mutation (g+6723G  >  A) introgression: comparative analysis of growth, slaughter, and carcass traits in Texel, Pırlak, and their crosses (F_1_ and BC_1_)

**DOI:** 10.5194/aab-67-207-2024

**Published:** 2024-05-03

**Authors:** Serdar Koçak, Mustafa Tekerli, Metin Erdoğan, Koray Çelikeloğlu, Ebubekir Yazıcı, Zehra Bozkurt, Özlem Hacan, Mustafa Demirtaş, Samet Çinkaya

**Affiliations:** 1 Department of Animal Science, Faculty of Veterinary Medicine, Afyon Kocatepe University, Afyonkarahisar, 03200, Türkiye; 2 Department of Veterinary Biology and Genetics, Faculty of Veterinary Medicine, Afyon Kocatepe University, Afyonkarahisar, 03200, Türkiye; 3 Department of Obstetrics and Gynecology, Faculty of Veterinary Medicine, Afyon Kocatepe University, Afyonkarahisar, 03200, Türkiye

## Abstract

This study was carried out to examine the effect of myostatin mutation on growth, body measurements, *Musculus longissimus dorsi* (MLD) values, and carcass characteristics in crossbred generations as well as introgression from Texel to Pırlak. This research was conducted on 105 F ${}_{1}$ , 94 myostatin-carrying BC ${}_{1}$ , 53 non-carrying BC ${}_{1}$ lambs, and pure Pırlak and Texel lambs born during the same period. It was determined that the effects of factors such as genotype, sex, birth type, birth month, and dam age were significant ( P< 0.05) for the growth characteristics of lambs. The birth weights, daily live weight gains and weaning weights in F ${}_{1}$ , myostatin-carrying BC ${}_{1}$ , and non-carrying BC ${}_{1}$ lambs were 4.10, 4.45, and 4.39, 0.21252, 0.22176, and 0.20964, as well as 35.51, 33.18, and 33.47 kg, respectively. It was detected that Texel and myostatin-carrying BC ${}_{1}$ lambs were significantly ( P< 0.05) higher than Pırlak and non-carrying BC ${}_{1}$ lambs for the MLD area at weaning. Additionally, the rump width, chest circumference, and MLD depth of lambs carrying myostatin mutation were significantly greater than Pırlak lambs. Six-month live weight means of Pırlak, Texel, and myostatin-carrying BC ${}_{1}$ lambs were found as 39.45, 37.22, and 39.06 kg, respectively. Myostatin-carrying BC ${}_{1}$ lambs had a superiority to Pırlak lambs in terms of muscle conformation and fatness of the hind leg.

As a result, myostatin-carrying BC ${}_{1}$ lambs were significantly found to have a better MLD area than non-carrying BC ${}_{1}$ lambs, indicating a myostatin mutation (g + 6723G > A) effect. It was concluded that the growth and carcass characteristics of Pırlak lambs may be improved by introgression of myostatin mutation.

## 1 Introduction

Milk, meat, and fleece production of sheep are considered important aspects of human life. Türkiye has 44–45 million sheep, with the majority representing native breeds (TUIK, 2023). The Pırlak breed is found intensively in the central Western Anatolia region in Türkiye. The growth rate and meat quality of native sheep breeds in Türkiye are moderate. It is necessary to expand the gene pools of native breeds to accomplish high fertility, rapid growth, and improved meat quality. The Texel breed is known for its double-muscled phenotype and meat quality. Clop et al. (2006) reported that the G-to-A transition in the 3 ${}^{\prime }$ untranslated region (UTR) of GDF8 allele (g + 6723G > A) is a major contributor to the muscular hypertrophy phenotype in Texel sheep. Lean muscle growth is an important trait for improving sheep productivity. The live weights and body measurements are important parameters of lamb growth. Birth weight, daily live weight gain, weaning weight, and 12-month live weight ranged from 3.36 to 4.76 kg, 147.51 to 318.00 g, 20.90 to 34.38 kg, and 37.6 to 46.7 kg, respectively, for purebred Texel lambs in the literature (McMillan et al., 1988; Khusro et al., 2005; Maxa et al., 2007; de Fatima Sieklicki et al., 2016; Armstrong et al., 2018; Çelikeloğlu et al., 2022). The values related to the same traits in Texel crosses vary between 3.04 and 5.30 kg, 170 and 267 g, 14.86 and 33.47 kg, and 50.41 and 51.40 kg, respectively (Wuliji et al., 1995; Freking and Leymaster, 2004; Ali et al., 2005; Koritiaki et al., 2013; de Vargas Junior et al., 2014; Çelikeloğlu et al., 2022; Tekerli et al., 2022). Also, Çelikeloğlu et al. (2022) reported 6-month live weights of 34.40 and 36.91 kg for F ${}_{1}$ and BC ${}_{1}$ Texel cross lambs. Pırlak lambs were found within the ranges of 3.18–3.42 kg, 23.32–25.69 kg, 160–183 g, 31.45–35.97 kg, and 38.09–43.87 kg for birth weight, weaning weight, daily live weight gain, and 6- and 12-month live weights (Tekerli et al., 2016). Ultra-sonographic muscle depth and area of lambs carrying the myostatin mutation were deeper and wider than those of non-carrying lambs according to Masri et al. (2011). Milerski et al. (2006) reported that the Texel lambs were leaner than the Suffolk and Romney breeds.

This study was carried out to evaluate the transfer of the myostatin mutation (g + 6723G > A) in the GDF8 gene from Texel to Pırlak (wild type) by introgression and to reveal the results for live weights, body measurements, ultrasound measures, and slaughter characteristics in Texel, Pırlak, and their F ${}_{1}$ and BC ${}_{1}$ cross lambs.

## 2 Material and methods

The animals were used in compliance with the rules of the experimental animals ethical committee at Afyon Kocatepe University (decision no. 49533702-47). This study was conducted on 105 F ${}_{1}$ , 94 myostatin-carrying, and 53 non-carrying BC ${}_{1}$ lambs born between 2016 and 2019 as well as Pırlak and Texel lambs born during the same period at the Afyonkarahisar Province Sheep and Goat Breeders Association's Stud Animals Breeding and Test Station. F ${}_{1}$ lambs were obtained from the mating of Pırlak ewes with homozygous myostatin-mutation-carrying Texel rams. Male lambs carrying the myostatin gene from this group were mated with Pırlak ewes, resulting in the birth of myostatin-carrying and non-carrying BC ${}_{1}$ lambs. Birth weights of lambs were taken in the first 24 h, and weaning weights were obtained with precision scales at about 120 d age. According to the selection program conducted on the farm, non-carrying BC ${}_{1}$ lambs were culled after weaning. The average daily live weight gain of lambs was calculated from birth to weaning. Six and 12 months of weights of studs were calculated by interpolation from the two measures taken before and after the mentioned date. Interpolations were done by using an in-home data recording program, Çoban Yıldızı. Wither height, body length, rump width, and chest circumference were assessed by measuring stick, compass, and tape at the weaning (Tekerli et al., 2022). In addition, ultra-sonographic *Musculus longissimus dorsi* (MLD) area, MLD depth, and subcutaneous fat thickness were recorded. The process was performed between the 12th and 13th ribs (Leeds et al., 2008) using a B-Mode real-time USG device (Mindray DP10, China) and a 5 MHz convex probe. The ultrasonic images were displayed and recorded with an MP4 player (Orite^®^ PMP500, Australia) and then captured on a computer. The records were monitorized with a GOM Player (Gretech Corporation, South Korea), and the images were prepared for measuring ultrasound parameters. These images were processed to determine the ultrasonic measures (Bracken et al., 2006) by using ImageJ software (National Institute of Health, Bethesda, USA). A total of 30 male lambs born as twins, consisting of 10 each from myostatin-carrying BC ${}_{1}$ lambs, pure Pırlak, and homozygous myostatin-carrying Texel (87.5 % Texel and 12.5 % Ramlıç) were slaughtered at approximately 24 weeks' age in an abattoir. The hot carcass, head, skin, foot, heart and lungs, gastrointestinal tract (both full and empty), liver, spleen, testicles, kidneys, and kidney fat were weighed. The commercial and real dressing percentages were calculated according to Tekerli et al. (2022). The pH and color parameters ( $L^{*}$ , $a^{*}$ , $b^{*}$ ) of the MLD were measured within 1 h after slaughter and 24 h later at + 4 °C using a WTW Multi 3410 SET 1 2FD451 pH meter and a Konica Minolta ChromaMeter CR-400 device. The cold carcass was divided in half along the spine. The carcass external–internal length, shoulder width, rump width, chest depth, hind leg length 1, and hind leg length 2 were determined in the right-half carcass using a measuring tape as described in Tekerli et al. (2022). The left-half carcass was split into seven parts (shoulder, belly, hind leg, neck, anterior rib, loin rib, and tail) according to the method reported by Colomer Rocher et al. (1988). The bone, muscle, and fat in the left hind leg were separated and weighed individually. The genetic analysis processes for detecting the myostatin mutation were conducted and interpreted as reported by Çelikeloğlu et al. (2022).

The statistical models below were used for the traits examined.

For birth weight and daily live weight gain,

$$Y_{\mathrm{ijklmn}}=\mu +G_{i}+S_{j}+{\text{BT}}_{k}+{\text{BM}}_{l}+{\text{DA}}_{m}+e_{\mathrm{ijklmn}}.$$

For weaning weight, body measurements, MLD traits, and 6- and 12-month live weight,

$$Y_{\mathrm{ijklmn}}=\mu +G_{i}+S_{j}+{\text{BT}}_{k}+{\text{BM}}_{l}+{\text{DA}}_{m}+\text{b1(BW)}+e_{\mathrm{ijklmn}}.$$

For slaughter and carcass traits,

$$Y_{\mathrm{ij}}=\mu +G_{i}+e_{\mathrm{ij}}.$$

The models were categorized as follows: genotype (Pırlak, F ${}_{1}$ , myostatin-carrying BC ${}_{1}$ , non-carrying BC ${}_{1}$ , Texel); sex (male and female); birth type (single and multiple); birth month (December and January); and dam age ( ≤ 24, > 24, ≤ 36, and > 36 months). Weaning and post-weaning body weights and body measurements were adjusted to 120 d (weaning age). Birth weights (BWs) were considered covariate (b1) for weaning weight, body measurements, and MLD traits and 6- and 12-month live weights in the statistical analysis. Variance analysis and multiple comparison tests were performed using the PASW Statistics software for Windows, version 18.0.0 (SPSS, Chicago, IL).

## 3 Results

The means and standard errors of live weights, daily live weight gain, body measurements, ultra-sonographic MLD area, MLD depth, and subcutaneous fat thickness for Pırlak, F ${}_{1}$ , myostatin-carrying BC ${}_{1}$ and non-carrying BC ${}_{1}$ , and Texel lambs are given in Tables 1, 2, and 3. Genotype, sex, birth type, and dam age significantly affected the birth weight ( P< 0.05). Significant factors affecting daily live weight gain were genotype, sex, birth type and month, and dam age. The highest daily live weight gain (221.76 g) was observed in myostatin-carrying BC ${}_{1}$ lambs. Weaning weights in F ${}_{1}$ , myostatin-carrying BC ${}_{1}$ , and non-carrying BC ${}_{1}$ lambs were 35.51, 33.18, and 33.47 kg, respectively. All factors except dam age had significant ( P< 0.05) effects on the trait in the 2016–2017 season. However, only sex and birth month had a significant effect on weaning weight in the 2018–2019 season. Genotype, sex, and birth month significantly affected post-weaning growth ( P< 0.05). All factors except dam age had significant ( P< 0.05) effects for body measurements, ultra-sonographic MLD area, and MLD depth of Pırlak, myostatin-carrying BC ${}_{1}$ , non-carrying BC ${}_{1}$ , and Texel lambs at the age of weaning.

**Table 1:** Least-squares means for growth traits of Pırlak, F ${}_{1}$ , and Texel lambs born in 2016–2017.

		Birth weight		Daily live weight gain		Weaning weight		Six-month		Twelve-month	
		(kg)		(kg)		(kg)		live weight		live weight	
								(kg)		(kg)	
		n	$\bar{X}\pm S\bar{x}$	n	$\bar{X}\pm S\bar{x}$	n	$\bar{X}\pm S\bar{x}$	n	$\bar{X}\pm S\bar{x}$	n	$\bar{X}\pm S\bar{x}$
	μ	378	4.32 ± 0.06	378	0.20270 ± 0.00368	378	32.97 ± 0.49	146	38.47 ± 0.54	132	50.20 ± 1.44
Genotype			${}^{***}$		${}^{*}$		${}^{***}$		${}^{***}$		${}^{***}$
	Pırlak	236	4.13 ± 0.06 ${}^{b}$	236	0.20398 ± 0.00360 ${}^{\mathrm{ab}}$ ​​​​​​​	236	33.53 ± 0.49 ${}^{b}$	66	40.75 ± 0.64 ${}^{a}$	61	57.38 ± 1.96 ${}^{a}$
	F ${}_{1}$	105	4.10 ± 0.08 ${}^{b}$	105	0.21252 ± 0.00459 ${}^{a}$	105	35.51 ± 0.62 ${}^{a}$	52	41.28 ± 0.73 ${}^{a}$	49	54.06 ± 1.76 ${}^{a}$
	Texel	37	4.71 ± 0.13 ${}^{a}$	37	0.19161 ± 0.00732 ${}^{b}$	37	29.87 ± 0.99 ${}^{c}$	28	33.39 ± 0.99 ${}^{b}$	22	39.16 ± 3.49 ${}^{b}$
Sex			${}^{***}$		${}^{***}$		${}^{***}$		${}^{***}$		${}^{***}$
	Male	178	4.43 ± 0.08 ${}^{a}$	178	0.21937 ± 0.00440 ${}^{a}$	178	34.94 ± 0.59 ${}^{a}$	62	41.69 ± 0.69	56	58.81 ± 1.81 ${}^{a}$
	Female	200	4.21 ± 0.07 ${}^{b}$	200	0.18603 ± 0.00406 ${}^{b}$	200	30.99 ± 0.54 ${}^{b}$	84	35.26 ± 0.63	76	41.59 ± 2.06 ${}^{b}$
Birth type			${}^{***}$		${}^{***}$		${}^{**}$		NS		NS
	Single	212	4.79 ± 0.07 ${}^{a}$	212	0.21844 ± 0.00389 ${}^{a}$	212	33.95 ± 0.55 ${}^{a}$	97	38.83 ± 0.57	91	52.86 ± 1.27
	Multiple	166	3.84 ± 0.08 ${}^{b}$	166	0.18696 ± 0.00460 ${}^{b}$	166	31.99 ± 0.64 ${}^{b}$	49	38.12 ± 0.83	41	47.54 ± 2.58
Birth month			NS		${}^{***}$		${}^{***}$		${}^{*}$		NS
	December	57	4.26 ± 0.10	57	0.21261 ± 0.00590 ${}^{a}$	57	35.39 ± 0.79 ${}^{a}$	33	39.59 ± 0.47 ${}^{a}$	30	50.30 ± 2.60
	January	321	4.37 ± 0.05	321	0.19280 ± 0.00307 ${}^{b}$	321	30.55 ± 0.41 ${}^{b}$	113	37.36 ± 0.47 ${}^{b}$	102	50.10 ± 1.09
Dam age (month)			${}^{*}$		NS		NS		NS		NS
	≤ 24	80	4.23 ± 0.09 ${}^{b}$	80	0.20173 ± 0.00548	80	32.98 ± 0.73	39	38.05 ± 0.89	36	46.06 ± 2.49
	24 < and ≤ 36	79	4.28 ± 0.10 ${}^{b}$	79	0.20408 ± 0.00553	79	33.47 ± 0.74	37	39.23 ± 0.85	34	52.99 ± 2.37
	> 36	219	4.44 ± 0.07 ${}^{a}$	219	0.20229 ± 0.00377	219	32.45 ± 0.51	70	38.14 ± 0.61	62	51.55 ± 1.96
Birth weight ${}^{†}$			–		–		${}^{***}$		${}^{***}$		NS

NS: non-significant. ${}^{*}$ P<0.05 ; ${}^{**}$ P< 0.01; ${}^{***}$ P< 0.001. ${}^{a,b,c}$ Different superscripts in the same column in each fixed effect are significantly different ( P<0.05 ). ${}^{†}$ The birth weights of lambs were fitted to the model as a covariate in weaning and post-weaning traits.

**Table 2:** Least-squares means for growth traits of Pırlak, myostatin-carrying BC ${}_{1}$ , non-carrying BC ${}_{1}$ , and Texel lambs born in 2018–2019.

		Birth weight		Daily live weight gain		Weaning weight		Six-month live weight	
		(kg)		(kg)		(kg)		(kg)	
		n	$\bar{X}\pm S\bar{x}$	n	$\bar{X}\pm S\bar{x}$	n	$\bar{X}\pm S\bar{x}$	n	$\bar{X}\pm S\bar{x}$
	μ	372	4.11 ± 0.08	372	0.20900 ± 0.00434	372	33.85 ± 0.57	190	38.58 ± 0.64
Genotype			${}^{***}$		${}^{**}$		NS		${}^{*}$
	Pırlak	169	3.81 ± 0.09 ${}^{b}$	169	0.20573 ± 0.00472 ${}^{\mathrm{ab}}$	169	33.82 ± 0.63	81	39.45 ± 0.71 ${}^{a}$
	Non-carrying BC ${}_{1}$	53	4.39 ± 0.12 ${}^{a}$	53	0.20964 ± 0.00640 ${}^{\mathrm{ab}}$	53	33.47 ± 0.85	–	–
	Carrying BC ${}_{1}$	94	4.45 ± 0.09 ${}^{a}$	94	0.22176 ± 0.00526 ${}^{a}$	94	33.18 ± 0.70	68	39.06 ± 0.70 ${}^{a}$
	Texel	56	3.79 ± 0.13 ${}^{b}$	56	0.19887 ± 0.00697 ${}^{b}$	56	34.95 ± 0.92	41	37.22 ± 0.92 ${}^{b}$
Sex			${}^{***}$		${}^{***}$		${}^{***}$		${}^{**}$
	Male	193	4.27 ± 0.09 ${}^{a}$	193	0.22710 ± 0.00488 ${}^{a}$	193	35.95 ± 0.64 ${}^{a}$	62	42.10 ± 0.76 ${}^{a}$
	Female	179	3.95 ± 0.09 ${}^{b}$	179	0.19090 ± 0.00471 ${}^{b}$	179	31.75 ± 0.62 ${}^{b}$	128	35.06 ± 0.68 ${}^{b}$
Birth type			${}^{***}$		${}^{***}$		NS		NS
	Single	145	4.54 ± 0.09 ${}^{a}$	145	0.21923 ± 0.00480 ${}^{a}$	145	34.23 ± 0.65	96	38.60 ± 0.67
	Multiple	227	3.68 ± 0.09 ${}^{b}$	227	0.19877 ± 0.00491 ${}^{b}$	227	33.48 ± 0.66	94	38.56 ± 0.77
Birth month			NS		${}^{***}$		${}^{***}$		${}^{***}$
	December	61	4.19 ± 0.11	61	0.22211 ± 0.00613 ${}^{a}$	61	36.52 ± 0.80 ${}^{a}$	42	40.06 ± 0.82 ${}^{a}$
	January	311	4.02 ± 0.07	311	0.19589 ± 0.00401 ${}^{b}$	311	31.18 ± 0.52 ${}^{b}$	148	37.10 ± 0.62 ${}^{b}$
Dam age (month)			${}^{*}$		${}^{*}$		NS		NS
	≤ 24	15	3.86 ± 0.19 ${}^{b}$	15	0.19573 ± 0.01031 ${}^{b}$	15	32.66 ± 1.35	6	38.97 ± 1.59
	24 < and ≤ 36	74	4.16 ± 0.09 ${}^{a}$	74	0.21142 ± 0.00524 ${}^{a}$	74	34.09 ± .069	33	38.49 ± 0.74
	> 36	283	4.31 ± 0.06 ${}^{a}$	283	0.21985 ± 0.00329 ${}^{a}$	283	34.80 ± 0.44	151	38.28 ± 0.38
Birth weight ${}^{†}$			–		–		${}^{***}$		${}^{***}$

NS: non-significant. ${}^{*}$ P<0.05 ; ${}^{**}$ P< 0.01; ${}^{***}$ P< 0.001. ${}^{a,b}$ Different superscripts in the same column in each fixed effect are significantly different ( P<0.05 ). ${}^{†}$ The birth weights of lambs were fitted to the model as a covariate in weaning and post-weaning traits.

**Table 3:** Least-squares means for weaning body measurements and MLD traits of Pırlak, myostatin-carrying BC ${}_{1}$ , non-carrying BC ${}_{1}$ , and Texel lambs born in 2018–2019.

		Wither height		Body length	Rump width	Chest	MLD area	MLD depth	Subcutaneous
		(cm)		(cm)	(cm)	circumference	(cm ${}^{2}$ )	(cm)	fat thickness
						(cm)			(cm)
		n	$\bar{X}\pm S\bar{x}$	$\bar{X}\pm S\bar{x}$	$\bar{X}\pm S\bar{x}$	$\bar{X}\pm S\bar{x}$	$\bar{X}\pm S\bar{x}$	$\bar{X}\pm S\bar{x}$	$\bar{X}\pm S\bar{x}$
	μ	269	60.09 ± 0.42	60.50 ± 0.55	14.21 ± 0.18	79.16 ± 0.77	9.496 ± 0.294	2.597 ± 0.054	0.587 ± 0.013
Genotype			${}^{***}$	${}^{***}$	${}^{***}$	${}^{***}$	${}^{***}$	${}^{***}$	${}^{**}$
	Pırlak	93	60.76 ± 0.52 ${}^{a}$	60.46 ± 0.67 ${}^{b}$	13.74 ± 0.22 ${}^{b}$	78.57 ± 0.95 ${}^{b}$	8.953 ± 0.362 ${}^{b}$	2.425 ± 0.066 ${}^{c}$	0.575 ± 0.016 ${}^{b}$
	Non-carrying BC ${}_{1}$	45	61.37 ± 0.53 ${}^{a}$	62.43 ± 0.69 ${}^{a}$	14.25 ± 0.22 ${}^{a}$	79.48 ± 0.97 ${}^{b}$	8.780 ± 0.370 ${}^{b}$	2.552 ± 0.068 ${}^{\mathrm{bc}}$	0.573 ± 0.016 ${}^{b}$
	Carrying BC ${}_{1}$	78	61.25 ± 0.46 ${}^{a}$	60.90 ± 0.60 ${}^{b}$	14.54 ± 0.20 ${}^{a}$	82.19 ± 0.85 ${}^{a}$	10.036 ± 0.323 ${}^{a}$	2.591 ± 0.059 ${}^{b}$	0.620 ± 0.014 ${}^{a}$
	Texel	53	56.97 ± 0.61 ${}^{b}$	58.20 ± 0.79 ${}^{c}$	14.31 ± 0.26 ${}^{a}$	76.40 ± 1.11 ${}^{c}$	10.214 ± 0.425 ${}^{a}$	2.821 ± 0.078 ${}^{a}$	0.581 ± 0.019 ${}^{b}$
Sex			${}^{***}$	${}^{***}$	${}^{***}$	${}^{**}$	${}^{*}$	${}^{*}$	NS
	Male	142	61.71 ± 0.46 ${}^{a}$	61.86 ± 0.60 ${}^{a}$	14.54 ± 0.20 ${}^{a}$	80.08 ± 0.85 ${}^{a}$	9.739 ± 0.323 ${}^{a}$	2.650 ± 0.059 ${}^{a}$	0.595 ± 0.014
	Female	127	58.47 ± 0.45 ${}^{b}$	59.13 ± 0.58 ${}^{b}$	13.88 ± 0.19 ${}^{b}$	78.24 ± 0.82 ${}^{b}$	9.252 ± 0.314 ${}^{b}$	2.545 ± 0.057 ${}^{b}$	0.579 ± 0.014
Birth type			${}^{***}$	${}^{*}$	${}^{**}$	${}^{**}$	${}^{***}$	${}^{***}$	${}^{***}$
	Single	112	60.72 ± 0.44 ${}^{a}$	61.11 ± 0.57 ${}^{a}$	14.43 ± 0.19 ${}^{a}$	80.87 ± 0.80 ${}^{a}$	10.075 ± 0.305 ${}^{a}$	2.686 ± 0.056 ${}^{a}$	0.613 ± 0.013 ${}^{a}$
	Multiple	157	59.46 ± 0.48 ${}^{b}$	59.88 ± 0.63 ${}^{b}$	13.99 ± 0.21 ${}^{b}$	77.45 ± 0.89 ${}^{b}$	8.917 ± 0.339 ${}^{b}$	2.508 ± 0.062 ${}^{b}$	0.561 ± 0.015 ${}^{b}$
Birth month			${}^{***}$	${}^{**}$	${}^{***}$	${}^{**}$	${}^{*}$	${}^{*}$	NS
	December	20	61.68 ± 0.71 ${}^{a}$	61.69 ± 0.93 ${}^{a}$	14.79 ± 0.30 ${}^{a}$	81.16 ± 1.31 ${}^{a}$	10.041 ± 0.499 ${}^{a}$	2.708 ± 0.091 ${}^{a}$	0.604 ± 0.022
	January	249	58.50 ± 0.30 ${}^{b}$	59.31 ± 0.39 ${}^{b}$	13.63 ± 0.13 ${}^{b}$	77.16 ± 0.54 ${}^{b}$	8.951 ± 0.207 ${}^{b}$	2.486 ± 0.038 ${}^{b}$	0.570 ± 0.009
Dam age (month)			NS	NS	NS	NS	NS	NS	NS
	≤ 24	15	59.90 ± 0.82	59.48 ± 1.06	14.27 ± 0.35	77.84 ± 1.49	9.299 ± 0.570	2.529 ± 0.104	0.581 ± 0.025
	24 < and ≤ 36	51	60.01 ± 0.50	60.80 ± 0.65	14.08 ± 0.21	79.16 ± 0.91	9.563 ± 0.350	2.577 ± 0.064	0.577 ± 0.015
	> 36	203	60.35 ± 0.36	61.21 ± 0.46	14.28 ± 0.15	80.48 ± 0.65	9.625 ± 0.248	2.686 ± 0.045	0.604 ± 0.011
Birth weight ${}^{†}$			NS	NS	NS	NS	NS	NS	NS

NS: non-significant. ${}^{*}$ P<0.05 ; ${}^{**}$ P< 0.01; ${}^{***}$ P< 0.001. ${}^{a,b,c}$ Different superscripts in the same column in each fixed effect are significantly different ( P<0.05 ). ${}^{†}$ The birth weights of lambs were fitted to the model as a covariate in body measurements and MLD traits.

The findings related to slaughter and carcass characteristics of Pırlak, Texel, and myostatin-carrying BC ${}_{1}$ lambs are presented in Tables 4 and 5. Commercial hot dressing percentages were 47.84 %, 51.07 %, and 48.44 % in Pırlak, Texel, and myostatin-carrying BC ${}_{1}$ lambs, while commercial cold dressing percentages were determined to be 46.33 %, 49.78 %, and 47.15 %. Significant ( P< 0.01) differences in genotypes regarding commercial hot and cold dressing percentages were observed. External and internal carcass lengths were measured as 73.40 and 64.35, 63.40 and 57.80, and 73.50 and 63.70 cm in Pırlak, Texel, and myostatin-carrying BC ${}_{1}$ lambs, respectively. The chest depths in Pırlak, Texel, and myostatin-carrying BC ${}_{1}$ lamb carcasses were 25.10, 23.35, and 25.25 cm, respectively, and there was a significant difference ( P< 0.001) among genotypes. The hind leg length 1 in Pırlak, Texel, and myostatin-carrying BC ${}_{1}$ lambs was measured as 39.60, 36.10, and 40.00 cm, while the hind leg length 2 was 32.50, 29.60, and 33.10 cm, respectively. Texel lambs were significantly behind the others in these characteristics. In the left-half carcass, the tail weights were found to be 0.264, 0.072, and 0.154 kg in Pırlak, Texel, and myostatin-carrying BC ${}_{1}$ lambs, respectively, and the differences between the genotypes were significant ( P< 0.001). The tail ratio in the left-half carcass also showed significant differences ( P< 0.001) among the genotypes. Hind leg muscle weights were 1.747, 2.169, and 1.883 kg in Pırlak, Texel, and myostatin-carrying BC ${}_{1}$ lambs, respectively, and the muscle ratios were 66.36 %, 74.53 %, and 69.51 %. The differences were significantly ( P< 0.001) in favor of Texel lambs concerning these characteristics. The fat weights in the hind leg were 0.312, 0.169, and 0.241 kg, and the fat ratios were 11.79 %, 5.50 %, and 8.62 % in Pırlak, Texel, and myostatin-carrying BC ${}_{1}$ lambs, respectively. Significant ( P< 0.001) differences between the genotypes were found for these characteristics. Carrying BC ${}_{1}$ lambs were in between the other two genotypes.

**Table 4:** Slaughter and carcass traits in Pırlak, myostatin-carrying BC ${}_{1}$ , and Texel lambs ( $\bar{X}\pm S\bar{x}$ ).

Traits		Pırlak	Myostatin-carrying BC ${}_{1}$	Texel	P
		( n= 10)	( n= 10)	( n= 10)	
Slaughter weight (kg)		32.350 ± 0.538	32.740 ± 1.518	32.020 ± 1.994	
Empty body weight (kg)		29.257 ± 0.587	29.532 ± 1.504	28.707 ± 1.842	
Hot carcass weight (kg)		15.477 ± 0.299	15.875 ± 0.794	16.422 ± 1.141	
Commercial hot dressing percentage (%)		47.841 ± 0.475 ${}^{b}$	48.435 ± 0.647 ${}^{b}$	51.074 ± 0.740 ${}^{a}$	${}^{**}$
Real hot dressing percentage (%)		52.941 ± 0.633 ${}^{b}$	53.814 ± 0.727 ${}^{b}$	57.021 ± 0.769 ${}^{a}$	${}^{***}$
Cold carcass weight (kg)		14.989 ± 0.924	15.458 ± 0.781	16.012 ± 1.127	
Commercial cold dressing percentage (%)		46.330 ± 0.427 ${}^{b}$	47.147 ± 0.615 ${}^{b}$	49.779 ± 0.733 ${}^{a}$	${}^{***}$
Real cold dressing percentage (%)		51.266 ± 0.548 ${}^{b}$	52.380 ± 0.664 ${}^{b}$	55.572 ± 0.737 ${}^{a}$	${}^{***}$
Head weight (kg)		2.110 ± 0.027 ${}^{a}$	2.013 ± 0.078 ${}^{\mathrm{ab}}$	1.821 ± 0.078 ${}^{b}$	${}^{*}$
Skin weight (kg)		2.385 ± 0.069	2.325 ± 0.147	2.239 ± 0.135	
Foot weight (kg)		0.823 ± 0.017	0.809 ± 0.029	0.757 ± 0.030	
Heart and lung weight (kg)		0.752 ± 0.027	0.790 ± 0.046	0.807 ± 0.024	
Gastrointestinal tract (full) (kg)		7.098 ± 0.235	7.214 ± 0.360	6.525 ± 0.424	
Gastrointestinal tract (empty) (kg)		4.005 ± 0.221	4.006 ± 0.352	3.212 ± 0.294	
Liver weight (kg)		0.559 ± 0.021	0.554 ± 0.030	0.613 ± 0.037	
Spleen weight (kg)		0.082 ± 0.003	0.087 ± 0.005	0.085 ± 0.007	
Testicles weight (kg)		0.292 ± 0.025	0.241 ± 0.027	0.252 ± 0.034	
Kidney and fat weight (kg)		0.184 ± 0.007	0.182 ± 0.013	0.185 ± 0.010	
pH	1 h	6.593 ± 0.051	6.664 ± 0.066	6.708 ± 0.086	
	24 h	5.071 ± 0.342	5.504 ± 0.210	5.485 ± 0.083	
L	1 h	31.726 ± 0.777	32.277 ± 0.464	33.115 ± 0.499	
	24 h	35.911 ± 0.886	36.415 ± 0.395	38.132 ± 0.718	
a	1 h	11.391 ± 0.229 ${}^{a}$	10.369 ± 0.345 ${}^{a}$	8.857 ± 0.299 ${}^{b}$	${}^{***}$
	24 h	14.397 ± 0.482	13.984 ± 0.641	12.984 ± 0.582	
b	1 h	3.185 ± 0.119	3.208 ± 0.220	2.931 ± 0.283	
	24 h	7.769 ± 0.292	7.802 ± 0.486	8.244 ± 0.278	

${}^{*}$ P<0.05 ; ${}^{**}$ P< 0.01; ${}^{***}$ P< 0.001. ${}^{a,b}$ Different superscripts in the same line are significantly different ( P<0.05 ).

**Table 5:** Carcass measurements and weights in Pırlak, myostatin-carrying BC ${}_{1}$ , and Texel lambs ( $\bar{X}\pm S\bar{x}$ ).

Traits	Pırlak	Myostatin-carrying BC ${}_{1}$	Texel	P
	( n= 10)	( n= 10)	( n= 10)	
External carcass length (cm)	73.400 ± 0.686 ${}^{a}$	73.500 ± 1.258 ${}^{a}$	63.400 ± 1.185 ${}^{b}$	${}^{***}$
Internal carcass length (cm)	64.350 ± 0.558 ${}^{a}$	63.700 ± 0.907 ${}^{a}$	57.800 ± 0.987 ${}^{b}$	${}^{***}$
Shoulder width (cm)	17.250 ± 0.593	17.100 ± 0.314	18.900 ± 0.781	
Rump width (cm)	20.000 ± 0.447	20.500 ± 0.500	21.000 ± 0.745	
Chest depth (cm)	25.100 ± 0.233 ${}^{a}$	25.250 ± 0.291 ${}^{a}$	23.350 ± 0.380 ${}^{b}$	${}^{***}$
Hind leg length 1 (cm)	39.600 ± 0.427 ${}^{a}$	40.000 ± 0.494 ${}^{a}$	36.100 ± 0.586 ${}^{b}$	${}^{***}$
Hind leg length 2 (cm)	32.500 ± 0.428 ${}^{a}$	33.100 ± 0.407 ${}^{a}$	29.600 ± 0.306 ${}^{b}$	${}^{***}$
Left-half carcass weight (kg)	7.801 ± 0.145	8.133 ± 0.405	8.337 ± 0.598	
Shoulder weight (kg)	1.468 ± 0.041	1.588 ± 0.074	1.621 ± 0.116	
Neck weight (kg)	0.668 ± 0.027	0.706 ± 0.042	0.656 ± 0.061	
Belly weight (kg)	0.690 ± 0.032	0.766 ± 0.057	0.807 ± 0.065	
Anterior rib weight (kg)	0.523 ± 0.024	0.558 ± 0.029	0.561 ± 0.041	
Loin rib weight (kg)	1.582 ± 0.041	1.631 ± 0.091	1.682 ± 0.125	
Tail weight (kg)	0.264 ± 0.023 ${}^{a}$	0.154 ± 0.020 ${}^{b}$	0.072 ± 0.009 ${}^{c}$	${}^{***}$
Hind leg weight (kg)	2.634 ± 0.043	2.712 ± 0.127	2.901 ± 0.213	
Shoulder ratio (%)	18.816 ± 0.376	19.599 ± 0.181	19.458 ± 0.217	
Neck ratio (%)	8.555 ± 0.307	8.674 ± 0.297	7.809 ± 0.391	
Belly ratio (%)	8.840 ± 0.357	9.337 ± 0.350	9.634 ± 0.225	
Anterior rib ratio (%)	6.687 ± 0.253	6.868 ± 0.144	6.732 ± 0.117	
Loin rib ratio (%)	20.275 ± 0.371	20.007 ± 0.300	20.175 ± 0.414	
Tail ratio (%)	3.361 ± 0.262 ${}^{a}$	1.855 ± 0.201 ${}^{b}$	0.841 ± 0.064 ${}^{c}$	${}^{***}$
Hind leg ratio (%)	33.810 ± 0.504	33.431 ± 0.492	34.763 ± 0.502	
Hind leg muscle weight (kg)	1.747 ± 0.034 ${}^{b}$	1.883 ± 0.088 ${}^{\mathrm{ab}}$	2.169 ± 0.168 ${}^{a}$	${}^{*}$
Hind leg bone weight (kg)	0.581 ± 0.012	0.596 ± 0.023	0.571 ± 0.025	
Hind leg fat weight (kg)	0.312 ± 0.026 ${}^{a}$	0.241 ± 0.034 ${}^{\mathrm{ab}}$	0.169 ± 0.028 ${}^{b}$	${}^{**}$
Hind leg muscle ratio (%)	66.360 ± 0.889 ${}^{c}$	69.509 ± 0.797 ${}^{b}$	74.533 ± 0.675 ${}^{a}$	${}^{***}$
Hind leg bone ratio (%)	22.053 ± 0.388	22.198 ± 0.824	20.197 ± 0.882	
Hind leg fat ratio (%)	11.789 ± 0.925 ${}^{a}$	8.615 ± 0.983 ${}^{b}$	5.501 ± 0.574 ${}^{c}$	${}^{***}$

${}^{*}$ P<0.05 ; ${}^{**}$ P< 0.01; ${}^{***}$ P< 0.001. ${}^{a,b,c}$ Different superscripts in the same line are significantly different ( P<0.05 ).

## 4 Discussion

In this study, while Texel lambs had a heavier birth weight than F ${}_{1}$ lambs, F ${}_{1}$ lambs were found to be superior to Texel lambs in terms of daily live weight gain, weaning weight, and 6- and 12-month live weights. The birth weight, daily live weight gain, and 6-month live weight averages of myostatin-carrying BC ${}_{1}$ lambs were higher than Texel lambs. The growth performances of the genotypes up to weaning in this study were in the range of values reported in the literature for pure Texel and crosses (McMillan et al., 1988; Wuliji et al., 1995; Freking and Leymaster, 2004; Ali et al., 2005; Khusro et al., 2005; Maxa et al., 2007; Koritiaki et al., 2013; de Vargas Junior et al., 2014; de Fatima Sieklicki et al., 2016; Armstrong et al., 2018; Çelikeloğlu et al., 2022; Tekerli et al., 2022). Genotype had an insignificant effect on weaning weight, and myostatin did not affect weaning weight either according to carrying and non-carrying lamb phenotypes. The 6-month live weight of myostatin-carrying BC ${}_{1}$ lambs was higher than the values of 34.40 and 36.91 kg reported for F ${}_{1}$ and BC ${}_{1}$ Texel crosses (Çelikeloğlu et al., 2022). The statistical analysis considering several environmental factors such as genotype, sex, and birth type showed that some environmental factors were significant for body weights. This is consistent with Tekerli et al. (2016).

The MLD area of myostatin-carrying BC ${}_{1}$ lambs showed a significant increase at weaning compared to non-carrying BC ${}_{1}$ and Pırlak lambs. A single copy of myostatin mutation had a significant impact of MLD area in BC ${}_{1}$ lambs. Furthermore, Masri et al. (2011) obtained similar results, which showed a significant effect of myostatin mutation on *Musculus longissimus lumborum* (MLL) area. Freking et al. (2018) also reported the impact of single-copy myostatin mutation on muscle conformation and fat deposition. The myostatin-carrying BC ${}_{1}$ lambs have a higher MLD area compared to non-carrying BC ${}_{1}$ lambs, as compatible with the reports of Çelikeloğlu et al. (2022) and Tekerli et al. (2022) for Ramlıç BC ${}_{1}$ and BC ${}_{2}$ lambs. The MLD depth of myostatin-carrying BC ${}_{1}$ lambs was lower than the values in the range of 2.818–3.618 cm reported for Ramlıç BC ${}_{1}$ and BC ${}_{2}$ crosses in the literature (Çelikeloğlu et al., 2022; Tekerli et al., 2022). Moreover, the MLD depth of myostatin-carrying BC ${}_{1}$ lambs was higher than the MLD value of 2.40 cm reported for pure Texel and the ultrasound MLL depth values in the range of 2.34–2.44 cm reported for Texel crosses by different researchers (Milerski et al., 2006; Macfarlane et al., 2009; Lambe et al., 2010). The effects of genotype, sex, birth type, and birth month were significant ( P< 0.05) for some body measurements and MLD values.

Though the effect of genotype was insignificant in hot and cold carcass weights, the dressing percentage was significantly ( P< 0.01) different among the genotypes. Meanwhile, findings for dressing percentages in myostatin-carrying BC ${}_{1}$ lambs were higher than the values reported by Koçak et al. (2016) and Tekerli et al. (2022) for Pırlak, Anatolian Merino, Anatolian Merino × Pırlak F ${}_{1}$ crossbred, and myostatin-carrying Ramlıç BC ${}_{2}$ lambs. These differences may have been caused by genotype, feeding, and selection. Statistically, there were no differences between genotypes in the weight of skin, foot, heart–lung, gastrointestinal tract (full–empty), liver, spleen, testicles, and kidney fat.

Meat pH is one of the most important quality parameters. The differences between genotypes for pH values measured at the 1st and 24th hours after slaughter were not significant. The pH value of myostatin-carrying BC ${}_{1}$ detected at the 24th hour was lower than the values reported by different researchers (Ekiz et al., 2009; Koçak et al., 2016; Tekerli et al., 2022) for Turkish Merino, Ramlıç, Kıvırcık, Chios, İmroz, Pırlak, Anatolian Merino × Pırlak F ${}_{1}$ , and Texel. As for the L , a , and b values indicating the meat color at 24 h, the myostatin-carrying BC ${}_{1}$ lambs were higher in L and a values but lower in b values than the myostatin-carrying Ramlıç BC ${}_{2}$ mentioned by Tekerli et al. (2022). The carcasses of myostatin-carrying BC ${}_{1}$ lambs could be interpreted as brighter, redder, and lighter yellow than the myostatin-carrying Ramlıç BC ${}_{2}$ lambs reported by Tekerli et al. (2022). However, Anatolian Merino × Pırlak F ${}_{1}$ lambs (Koçak et al., 2016) had higher a and b but lower L values than our study.

Carcass measurements (internal carcass length and rump width) of myostatin-carrying BC ${}_{1}$ lambs were in the range of Pırlak and Texel lambs. Tekerli et al. (2022) noted lower values for the traits in the myostatin-carrying Ramlıç BC ${}_{2}$ lambs. As expected, the weights of different carcass cuts (shoulder, belly, hind leg, anterior rib, loin rib, and tail) and myostatin-carrying BC ${}_{1}$ lambs were also in the midst of Pırlak and Texel. Muscle and fat ratios in the hind leg were significant among the genotypes, and myostatin-carrying BC ${}_{1}$ lambs were better than pure Pırlak lambs.

## 5 Conclusions

The results indicated that the genotype, sex, birth type, birth month, and dam age significantly affected the growth performance. MLD traits of lambs were significantly affected by genotype, sex, birth type, and month. As a result, the myostatin effect has emerged in myostatin-carrying BC ${}_{1}$ lambs with better performance for the MLD area compared to non-carrying BC ${}_{1}$ lambs. The MLD area and rump width of myostatin-carrying BC ${}_{1}$ lambs were similar to Texel lambs and superior to Pırlak lambs. Myostatin-carrying BC ${}_{1}$ lambs were greater than Pırlak lambs in terms of muscle conformation and fatness of the hind leg. Introgression of myostatin mutation from Texel to Pırlak sheep may be able to improve the growth and carcass characteristics of Pırlak lambs. It would be beneficial for further studies to explore the effect of a double copy of myostatin.

## Data Availability

The data presented in this study are available from the corresponding author upon reasonable request.
